# Increased crevassing across accelerating Greenland Ice Sheet margins

**DOI:** 10.1038/s41561-024-01636-6

**Published:** 2025-02-03

**Authors:** Thomas R. Chudley, Ian M. Howat, Michalea D. King, Emma J. MacKie

**Affiliations:** 1 Department of Geography, Durham University, Durham, UK; 2 Byrd Polar and Climate Research Center, Ohio State University, Columbus, OH USA; 3 School of Earth Sciences, Ohio State University, Columbus, OH USA; 4 Polar Science Center, Applied Physics Laboratory, University of Washington, Seattle, WA USA; 5 Department of Geological Sciences, University of Florida, Gainesville, FL USA

**Keywords:** Cryospheric science, Climate-change impacts, Climate change

## Abstract

Surface crevassing on the Greenland Ice Sheet is a large source of uncertainty in processes controlling mass loss due to a lack of comprehensive observations of their location and evolution through time. Here we use high-resolution digital elevation models to map the three-dimensional volume of crevasse fields across the Greenland Ice Sheet in 2016 and 2021. We show that, between the two years, large and significant increases in crevasse volume occurred at marine-terminating sectors with accelerating flow (up to +25.3 ± 10.1% in the southeast sector), while the change in total ice-sheet-wide crevasse volume was within measurement error (+4.3 ± 5.9%). The sectoral increases were offset by a reduction in crevasse volume in the central west sector (−14.2 ± 3.2%), particularly at Sermeq Kujalleq (Jakobshavn Isbræ), which exhibited slowdown and thickening over the study period. Changes in crevasse volume correlate strongly with antecedent discharge changes, indicating that the acceleration of ice flow in Greenland forces significant increases in crevassing on a timescale of less than five years. This response provides a mechanism for mass-loss-promoting feedbacks on sub-decadal timescales, including increased calving, faster flow and accelerated water transfer to the bed.

## Abstract (web summary)

Greenland-wide observations of crevasse volume and distribution suggest substantial increases in crevassing between 2016 and 2021 at marine-terminating sectors with accelerating ice flow.

## Main

Surface crevasses result from spatial and temporal ice flow variability and, thus, are ubiquitous across the complex, fast-flowing margins of the Greenland Ice Sheet (GrIS). Crevasses exert a first-order control on varied glaciological processes: fractures can act as pre-existing weaknesses that can promote calving and instability at glacier fronts^1^, while accumulated damage can soften the large-scale rheology of ice^2^. As key hydrological pathways^3–5^, crevasses transfer up to half of Greenland’s seasonal surface runoff to the bed^6^. This transport can alter ice rheology by increasing ice temperature^7^, modify the pressure of the subglacial hydrological system^4,5,8,9^ and promote basal melt^10^. By modulating the rate of meltwater transport to the ocean, further influence is exerted on terminus melt, fjord circulation and fjord biogeochemistry^11–13^. These crevasse-dependent processes hold the potential to induce substantial feedbacks between ice flow acceleration and mass loss^4,14^, making them a key source of uncertainty in projections of future GrIS behaviour^1,15^.

Given these mass-loss-accelerating feedbacks, it is critical to understand how crevasse fields are changing across Greenland. It is expected that increases in crevasse extent are common across the ice sheet due to (1) increasing tensile stresses resulting from a steepening ablation area and outlet glacier acceleration^14^; and (2) an increase in meltwater available for hydrofracture^9^. Only one multitemporal study exists, which observed an increase in crevasse extent across a region of West Greenland between 1985 and 2009^4^. However, observations of surging glaciers have shown that crevasse fields can propagate on much faster timescales (months to years) in response to rapid dynamic change^16,17^. Outlet glaciers around the GrIS are exhibiting accelerations of the same magnitude and rate as glacier surges^18–20^, suggesting that recent accelerations could initiate crevasse growth and subsequent feedbacks over sub-decadal timescales. However, studies monitoring short-term change in crevassing in Greenland and comprehensive assessments across the full ice sheet are lacking.

Recognition of the importance of crevassing has motivated improved observation and modelling capabilities. Studies have shown that simple parameterizations used in modelling studies are not a good predictor of crevasse distribution^3,21^ due to mixed-mode fracture formation^22^, variable ice rheology^23^ and the advection of crevasses from zones of active opening^24^. Therefore, improved observations are required to develop and validate models of fracture formation and propagation^25^, and parameterize their behaviour in models of ice-sheet dynamics and hydrology^6,26^. Satellite observation methods have progressed from manual delineation^4^ to computer vision^27,28^ and machine learning^29,30^ approaches. However, these are limited to assessing crevasse presence without critical information about crevasse depth, and attempts to map geometry have thus far been limited to profiles^21^. Recent public availability of comprehensive, multitemporal and high-resolution digital elevation models (DEMs) of the polar regions^31^ provide an unprecedented opportunity to assess three-dimensional (3D) crevasse geometry and evolution at high spatial and temporal resolution. Here we use these data to present a 3D record of crevassing over the entire GrIS in 2016 and 2021, across a period of time with notable dynamic accelerations^18–20^ and decelerations^32^. We use these maps to quantify the rate and extent of regional trends in crevassing and provide ice-sheet-wide observational evidence of the relationship between crevassing and ice dynamic change.

## Multitemporal Greenland-wide crevasse inventories

**Fig. 1:**
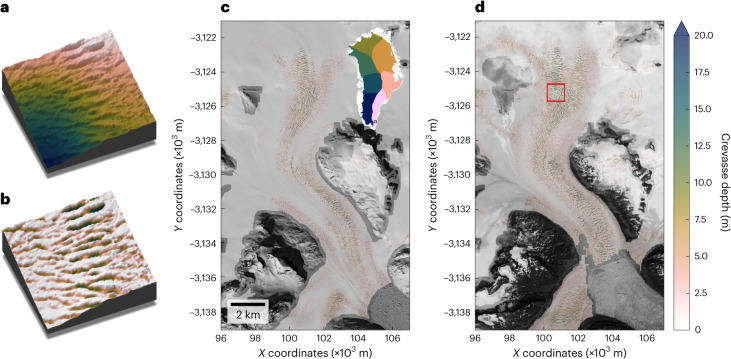
Examples of crevasse field extraction and evolution from ArcticDEM strips. **a**, A 500 × 500 m ArcticDEM sample of a crevassed surface, shaded from lowest (darkest) to highest (lightest) elevation. **b**, Sample following crevasse extraction, with a colour scale matching **c** and **d**. **c**, Crevasse depths at the head of Anorituup Kangerlua fjord from a 13 April 2016 ArcticDEM strip, overlaid onto a contemporaneous Worldview-1 image. Inset: location of Anorituup Kangerlua fjord (white box) within Greenland, with sectors as defined by ref. ^50^ coloured to match Figs. 2 and 3. **d**, Same as **c**, but for 15 July 2021 after sustained acceleration and retreat. Red box identifies regions of **a** and **b**. Panels **c** and **d** coordinates are in polar stereographic north (EPSG:3413).

**Fig. 2:**
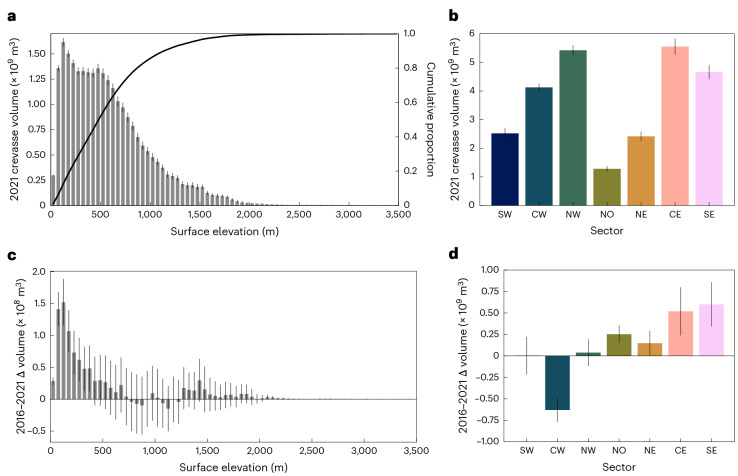
Crevasse volume and changes across the ice sheet. **a**, Histogram of 2021 crevasse volume with surface elevation across the ice sheet. **b**, Bar chart of 2021 crevasse volume. Bars indicate total crevasse volume per sector. **c**, Histogram of 2016–2021 crevasse volume change with surface elevation across the ice sheet. **d**, Bar chart of 2016–2021 crevasse volume change. Bars indicate change in total crevasse volume per sector, and are coloured to match Figs. 1 and 3. Error bars represent 2*σ* measurement uncertainties (Methods). A version of Fig. 2d with the ice-sheet-wide value presented for scale is included as Supplementary Fig. 1.

**Extended Data Fig. 1:**
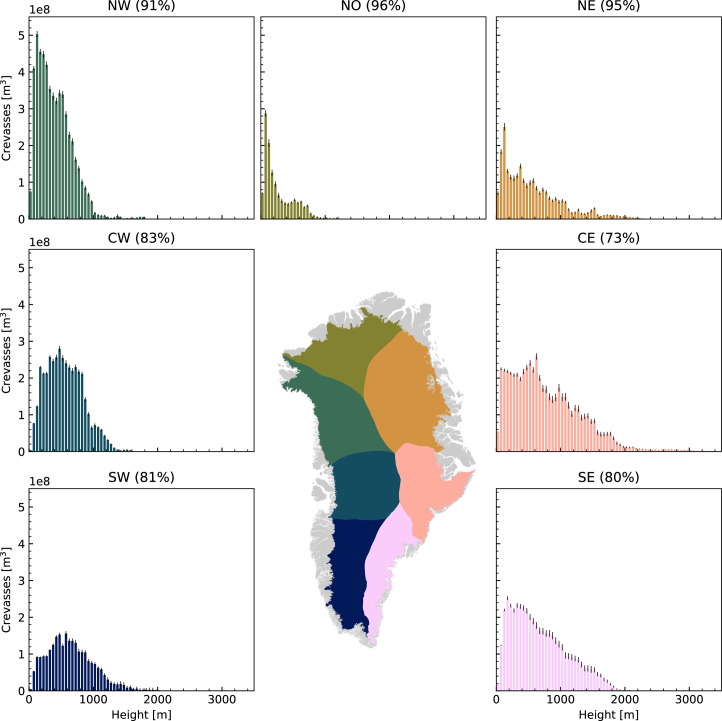
Histograms of sectoral crevasse volume in 2021 by surface elevation. Bars indicate total crevasse volume per 50 m surface elevation band per sector. Error bars represent 2σ uncertainties (Methods). Percentages in figure headings represent proportional data coverage of sector.

We extracted crevasse depth from 2 m resolution ArcticDEM strips^31^ across the GrIS in 2016 and 2021 (Fig. 1 and Methods). We integrated pixel-based crevasse depth to estimate the air-filled crevasse volume, providing estimates of crevasse inventory and change at an ice sheet, sector and basin scale. We mapped an estimated 25.98 × 10^9^ ± 1.30 × 10^9^ m^3^ of crevasse volume across ~89% of the melt zone (Methods) of the GrIS in 2021. Crevasse distribution overwhelmingly dominated low elevations near the ice margin (Fig. 2a), with 68% of crevasse volume concentrated below 700 m above mean sea level (AMSL) and 95% below 1,420 m AMSL. However, crevasses were less present at the lowest elevations, below 100 m AMSL (Fig. 2a), mostly due to the height of marine-terminating ice cliffs^33^. Hence, beneath 100 m, marine-terminating crevasses are limited by ice cliff height, or ice is land-terminating without substantial crevassing. Significant sectoral variation was observed (Fig. 2b and Supplementary Table 1), with high volumes of crevasses in the central east (CE), northwest (NW), southeast (SE) and central west (CW) sectors (typified by large, fast-flowing marine outlets), and lower volumes in the land-terminating southwest (SW) and less-dynamic north (NO) and northeast (NE) sectors. The crevasse elevation distribution was also highly variable between sectors (Extended Data Fig. 1). Sector NW exhibited a sharp elevation gradient in crevasse volumes, up to 1,000 m AMSL, while the similarly marine-terminating SE and CE sectors had longer-tailed distributions up to 2,000 m AMSL. This reflects the typical long trunks of SE/CE sectors, which extended diffusive acceleration from the ice front along their length, while NW glaciers are closely linked to the surrounding ice sheet with strongly convergent flow until close to the glacier margins^34,35^. Sectors NO and NE are characterized by a low-elevation bias, with little crevassing above 150 m. This probably reflects the predominance of crevassing on floating ice tongues concentrated in these sectors^36^. Finally, the unique distribution of sector CW, with the bulk of crevassing between the 200–800 m AMSL elevation bands, reflects the dominance of large marine-terminating outlets with short trunks and high calving fronts such as Sermeq Kujalleq (Jakobshavn Isbræ; SKJI).

**Extended Data Fig. 2:**
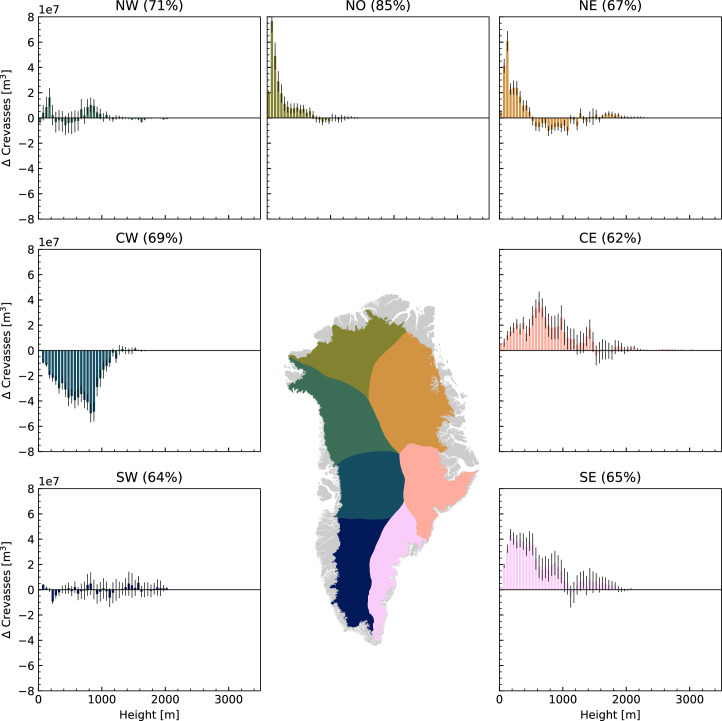
Histograms of sectoral crevasse volume change between 2016–2021 by surface elevation. Bars indicate change in total crevasse volume per 50 m surface elevation band per sector. Error bars represent 2σ uncertainty (Methods). Percentages in figure headings represent proportional data coverage of sector.

The change in crevasse volume from 2016 and 2021 across the GrIS was within measurement uncertainty, with a total change in crevasse volume of +9.32 × 10^8^ ± 13.01 × 10^8^ m^3^ (+4.3 ± 5.9%). However, this total masks spatially heterogeneous behaviour by elevation and sector. Beneath 400 m AMSL, crevasse volume increased significantly across all elevations, peaking at 100–150 m AMSL (Fig. 2c). Beneath ~100 m AMSL, increased crevassing was offset by a loss of surface area as marine-terminating glaciers retreated. Changes were highly heterogeneous at a sectoral level (Fig. 2d and Supplementary Table 1), varying between +25.3 ± 10.1% (NO) and −14.2 ± −3.2% (CW). No significant changes were observed in the NW, nor the land-terminating SW, while significant increases in the NO, NE, CE and SE were offset by a large reduction in the volume of crevasses in the CW sector (Fig. 2b). Sectors displayed distinct elevation distributions (Extended Data Fig. 2). In the NO and NE, increases were limited to ice tongues at the lowest elevations (<~400 m AMSL), while increases in the CE and SE were distributed more evenly across the lowest ~1,000 m AMSL due to diffusive thinning along the trunk.

## Relationship to dynamics

**Fig. 3:**
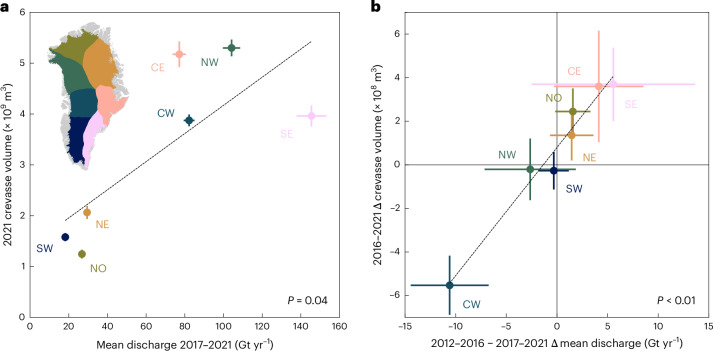
Sectoral scale discharge comparison. **a**, Scatter plot showing sectoral scale relationships between 2017–2021 mean annual discharge and 2021 crevasse volume. Error bars represent 2*σ* uncertainties (Methods). **b**, Scatter plot showing sectoral scale relationship between change in mean annual discharge between the 2011–2016 and 2017–2021 periods and change in crevasse volume between 2016 and 2021. Error bars represent 2*σ* uncertainties (Methods). Scatter plots and inset in **a** are coloured by sector to match Figs. 1 and 2. Note that only drainage basins with *>*60% crevasse observations and valid discharge records are included in the sectoral sum totals. Full regression results are presented in Supplementary Table 2.

Changes in crevasse morphology and extent reflect changes in ice dynamics: specifically, the surface stress regime^1,37,38^. We used records of total ice flux through outlet glacier termini, termed discharge, as a proxy for the bulk dynamic change of ice sectors and basins. Specifically, we compared annual crevasse volume (2021) with the mean discharge of the preceding five years (2017–2021), assuming that total crevasse volume in any individual year is the cumulative product of stresses integrated over multiple years (Methods). This proposed relationship between antecedent discharge and crevasse volume holds at a sectoral scale in our dataset (Fig. 3a; *P* = 0.04). Sectors predominantly comprising slow-flowing, land-terminating margins (SW) or less-dynamic, well-buttressed outlet glaciers (NO/NE) exhibited low crevasse volumes compared with sectors with high numbers of fast-flowing marine-terminating outlets (SE/CE/NW/CW).

We found a striking, sector-scale relationship (Fig. 3b; *P* < 0.01) between the change in crevasse volumes between 2016–2021 and the change in the corresponding antecedent five-year mean discharge (between 2012–2016 and 2017–2021), consistent with the hypothesis that changes in crevasse volume and extent are forced by changes in the dynamic regime of glaciers. Indeed, our large-scale crevasse observations closely parallel the Greenland discharge literature: both quantities are observed to exhibit insignificant/stable changes at an ice-sheet scale in the latter half of the 2010s, but this net figure masks significant inter-sectoral variation^39,40^. In particular, increases at eastern marine-terminating sectors are balanced by well-documented reductions in discharge from the CW sector in the second half of the 2010s^40^, mirroring the similar sectoral imbalance in crevasse volume change. This is largely driven by SKJI, which exhibited notable slowdown and thickening from 2016 to 2019, coinciding with cooler ocean temperatures^32^. Meanwhile, increased crevassing across the CE and SE sectors was consistent with accelerating ice velocities and discharge observed at both glacier and sectoral levels, linked to warming air and ocean temperatures^18,19,41,42^.

**Fig. 4:**
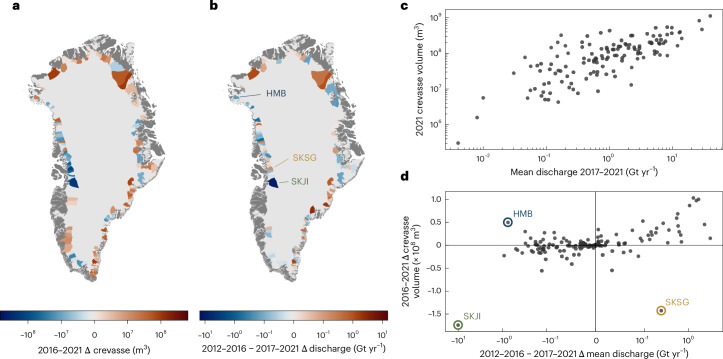
Basin-scale discharge comparison. **a**, Change in crevasse volume between 2016 and 2021 at basins with data coverage above the defined inclusion threshold (>60%). **b**, Change in mean annual discharge between the 2011–2016 and 2017–2021 periods. HMB, Harald Moltke Bræ; SKSG, Sermeq Kujalleq (Store Glacier); SKJI, Sermeq Kujalleq (Jakobshavn Isbræ). **c**, Basin-scale relationship between 2017–2021 mean annual discharge and 2021 crevasse volume. **d**, Basin-scale relationship between change in mean annual discharge between the 2011–2016 and 2017–2021 periods, and change in crevasse volume between 2016 and 2021. Outliers HMB, SKJI and SKSG are labelled. Only basins of a total area *>*100 km^2^ are shown.

We further assessed crevasse volume and changes at a basin level (Fig. 4a,b). This analysis confirmed a significant positive relationship (*P* < 0.01) between discharge and crevasse volume (Fig. 4c). This relationship exhibits a higher variability than the sectoral scale. We suggest that this relationship is again analogous to the Greenland discharge literature, whereby large-scale forcing is modulated by glacier-specific factors including, among others, fjord and glacier geometry^43^. In our case, local factors modulating the relationship between discharge and crevasse expression may include ice rheology (ice temperature, pre-existing damage and so on), the specific distribution of stresses (for example, plug flow concentrating high surface stresses into shear margins), and other factors including ice velocity, thickness and basal traction.

More nuance is revealed in the relationship between change in discharge and change in crevassing (Fig. 4d). Although there was a significant relationship between an increase in discharge and an increase in crevassing (*P* < 0.01 where Δ discharge > 0), there appeared to be a weaker relationship between crevassing and a decrease in discharge: in fact, the only glacier to display a notable reduction in both discharge and crevassing was SKJI. After excluding SKJI, the relationship was not significant (*P* = 0.44 where Δ discharge < 0). We suggest this could relate to differing timescales required to open and close crevasse fields, consistent with previous work that has concluded that crevasse formation outpaces crevasse closure^37,44^. Opening of crevasse fields probably occurs rapidly (<5 years), forced by the higher tensile surface stresses occurring alongside ice acceleration. However, an equivalent reduction in velocity at outlet glaciers does not necessitate a compressive stress regime that would actively close crevasses. Instead, the closure of crevasse fields requires the generational replacement of individual crevasses within a field by smaller crevasses formed under lower-tensile-stress conditions. As such, any reduction in crevasse field volume is rate-limited by surface velocity. The reduction in crevasse volume shown here at SKJI (labelled in Fig. 4d) may be an instructive exception, demonstrating how the fast-flowing regime propagated crevasse closure within a five-year timescale. Alternatively, the slowdown at SKJI after 2016^32^ could have induced sufficiently large regions of compression to actively close crevasses on a short timescale. While our work supports previous field observations that crevasse response to dynamics operates over multi-annual timescales^44^, the basin and sectoral scale heterogeneity observed here suggests that further work is necessary to understand response time variability and its controls.

Further individual basin-level anomalies also provide insights into crevasse behaviours. For instance, Harald Moltke Bræ (Fig. 4d) showed distinct reduction in discharge yet an increase in crevassing. This was an aliasing effect related to the surge occurring 2013–2019 (ref. ^45^), which resulted in an increase in (relict) crevasses between 2016 and 2021 even as the discharge reduced. Sermeq Kujalleq (Store Glacier; SKSG; Fig. 4d) exhibits the opposite anomaly, undergoing decreases in crevasse volume despite an increase in discharge. We hypothesize that this may relate to rapid summer deceleration events that occurred in 2018 and 2019 (Supplementary Fig. 2). SKSG consistently displays these behaviours, probably associated with instabilities in basal hydrology and sliding^46,47^. However, the deceleration events in these two summers were particularly extreme, with velocity collapsing by as much as 50% in 2019 (Supplementary Fig. 2). The resulting perturbation to the glacier strain field may have contributed to a reduced crevasse volume. If these seasonal deceleration events were contributory factors, the magnitude and variability of deceleration events may have an outsized impact on crevasse evolution in glaciers that exhibit this behaviour.

## Implications

We provide Greenland-wide observations of crevasse volume and distribution, revealing substantial changes in crevassing from 2016 to 2021 (sectoral scale variation from −14.2% to +25.3%) that correlate with the dynamic evolution of marine-terminating outlets. Although total change (+4.3 ± 5.9%) is within measurement uncertainty, significant sector-scale increases in crevassing occur in most sectors (Fig. 2d), offset by the CW sector—in particular SKJI, which is known to have undergone slowdown and thickening between 2016 and 2019^32^. Recent data indicate that SKJI is once again exhibiting acceleration and associated dynamic thinning^48^, suggesting that SKJI will no longer offset Greenland-wide increases in crevassing over the next few years. The five-year time step assessed here provides evidence of crevasse response time to dynamic changes in Greenland an order of magnitude faster than previously identified by satellite observation^4^. However, it is apparent that crevasse fields responded to dynamic events on a range of multi-annual timescales—in particular, slower responses where glaciers slowed—and further work should attempt to better clarify this response rate.

The ability to observe crevasses in 3D provides a major advance over 2D mapping from imagery alone^27,28^. We have observed significant increases in crevasse volume in pre-existing crevasse fields at low elevations (marine-terminating outlets). This change, not previously able to be assessed, highlights a pathway for externally forced (ocean- or atmosphere-driven) dynamic accelerations to generate a number of positive feedbacks to ice loss through increased crevassing^37^. Increased damage over annual timescales can act to weaken shear margins^2^. By transferring water to the bed^4,6,26^, crevasses induce rheological changes^7,14^, modify basal friction^4^ and—on reaching the ocean—amplify submarine melting at the terminus^13^. Finally, crevasses advected to the calving front play a role in accelerating glacier calving^1,49^. The ice-sheet-wide methods, datasets and behaviours presented here provide a starting point to properly calibrate and validate damage representation in large-scale dynamic models, accommodating the effects of ice damage and crevassing into predictions of future ice-sheet behaviour.

## Methods

### Crevasse detection

#### Crevasse depth detection from ArcticDEM strips

We mapped crevasses using 2 m resolution ArcticDEM v4.1 strips^31^ provided by the Polar Geospatial Center (PGC). The method, which we make public as a Python package and associated Jupyter Notebooks (https://github.com/trchudley/crevdem), will also work on other 2 m strips provided by the PGC as part of the REMA^51^ or EarthDEM^52^ projects, although we cannot guarantee the optimal length scale we determine here is representative of other sectors of the cryosphere. We first pre-processed the strips by filtering them only to good-quality ice surfaces. This was done by filtering strips to ‘good’ data as indicated by the PGC-provided bitmasks; filtering out bedrock using the Greenland Ice Mapping Project ice and ocean classification mask^53^; and geoid-correcting the heights to mean sea level using the EIGEN-6C4 geoid model^54^ provided within BedMachine v4^55^. Finally, when more than 1 km^2^ of strip area is <10 m AMSL, we applied a routine to filter out ‘marine surfaces’ (ocean, sea ice and low-lying ice mélange) following a previously published iceberg detection routine^56^. In this approach, we constructed a histogram of elevation in 0.25 m bins between −15 and +15 m AMSL, and identified contemporaneous sea level as the modal bin. We assigned all regions beneath 10 m of our determined contemporaneous sea level as marine surfaces, leaving only terrestrial ice and floating ice tongues.

After pre-processing, we determined the observed open-air crevasse depth, which we define here as the difference between the raw DEM height and a nominal ‘filled crevasse’ surface. We first detrended the DEM using a large Gaussian filter (200 m s.d.), before applying a black top hat (BTH) filter to the detrended surface to determine the negative deviation from the local maxima^57^. Gaussian and BTH filters were both applied using OpenCV implementations^58^. The diameter of the BTH kernel was set to be 60 m, following spatial variogram analysis of crevassed surfaces around Greenland (see section ‘Determining the optimal crevasse length scale’). Following previous approaches^57^, we identify pixels as ‘crevassed’ where the BTH-filtered value is greater than a threshold value, here >1 m. To generate a nominal ‘crevasse-filled’ surface, we further removed the crevassed pixels and filled the surfaces using an inverse-distance weighting algorithm as implemented in GDAL^59^, followed by two 3 × 3 averaging filter smoothing operations to dampen artefacts. Crevasse depth was determined as the difference between the interpolated ‘surface’ and the crevasse bottom in the raw DEM.

#### Determining the optimal crevasse length scale

To determine the kernel size, we assessed the typical crevasse length scale by modelling the spatial covariance, or variogram, which quantifies the variance of spatial measurements as a function of their separation distance^60^. The variogram was used to determine the range, or separation distance at which measurements are spatially uncorrelated. This parameter has previously been used to determine the optimal kernel size for BTH filtering of DEMs^57^. To find a representative range parameter, we estimated the ranges at four different glaciers covering a range of sectors and dynamic contexts: SKJI, SKSG, KJV Steenstrups Nordre Bræ and Isunnguata Sermia. We manually identified five 1,500 × 1,500 m sample zones, which we subjectively ranked on an ordinal scale of ‘crevasse intensity’ from 0 (no crevasses) to 4 (most crevassed region of glacier). We then constructed spatial variograms of the five sample zones using SciKit-GStat^61^. We used DEMs from 2021 (Supplementary Fig. 3a–d), which we detrended as described above, randomly sampling 2% of the pixels within the sample zone to increase computational efficiency. To estimate the representative crevasse width, we used the range of the variograms estimated using a Gaussian variogram model, which best fitted our experimental variograms. The mean estimated spatial range of the most crevassed sample regions (crevasse intensity = 4) was 62.4 m; the mean estimated spatial range of the top two most crevassed regions (crevasse intensity ≥ 3) was 57.3 m (Supplementary Fig. 3a–d). We selected 60 m as a representative range (and thus kernel size) to apply to fast-flowing regions of the GrIS.

#### Ice-sheet-wide processing and mosaicking

We produced GrIS-wide maps of crevasses in 2016 and 2021, years when ArcticDEM strip coverage was high and particularly conducive to comprehensive assessment.

To eliminate extraneous processing in the ice interior, we generously defined an area of interest mask as anywhere melt occurs in the RACMO2.3p2 1 km melt model between 2016 and 2021^62^, dilated by 10 km. We took all strips intersecting this region between April and October with a reported root mean square error <2 m and a component image baseline <60 minutes. In total, we processed 4,667 strips in 2016 and 4,207 strips in 2021 (Supplementary Table 3), with a subsequent coverage of our area of interest of 75% and 86% respectively (Supplementary Fig. 4). We note that coverage is biased towards outlet glaciers and no-data regions are commonly high-elevation, low-velocity sectors in the accumulation zone. This benefits our assessment as no-data regions are largely regions without crevassing present.

Owing to the advection of individual crevasses, 2 m resolution crevasse depth maps cannot be directly compared. Instead, we enabled comparison between 2016 and 2021 by summing crevasse depth maps into 200 m resolution crevasse volume maps, which we refer to as the ‘exposed crevasse air volume’. To obtain a single annual mosaic, we found the median value of all overlapping strips where multiple exist. All crevasse volumes discussed in this study have been aggregated into established sectors and basins^50^. However, for the interested reader, we present samples of changes at select basins at native resolution, alongside contemporaneous changes in the MEaSUREs Greenland annual ice-sheet velocity mosaics^63,64^, in Supplementary Fig. 5a–f.

#### Uncertainty and method intercomparison

We assigned a measurement uncertainty to our aggregate crevasse volume measurements by assessing variation in contemporaneous strip measurements. To do this, we assessed variance within the Nioghalvfjerdsfjorden (79° N) discharge basin in 2021, which we selected due to its high overlapping strip records (up to 21 overlapping strips) and large variation in surface types. Across all valid pixels within the 79° N area of interest, we calculated the per-pixel standard deviation in crevasse depth values across the basin. The mean standard deviation value across the 200 m grid cells was 407 m^3^ (10,175 m^3^ km^−2^). We apply this per-pixel uncertainty value to all basins, and present measurement uncertainty as 2*σ* error bars within the figures presented in this paper.

As a first-order comparison against alternative crevasse detection methods, we compare our method to contemporaneous crevasse datasets at a previously studied crevasse field (70.5399° N, 50.1423° W) located at SKSG in 2018. Here, there exists an uncrewed aerial vehicle (UAV)-derived 15 cm resolution map of crevasses (dated 8 July 2018) classified using object-based machine learning techniques^3^. We compare this against a Sentinel-2-derived map of crevasses using a Gabor filter approach^28^ for the date 2 July 2018, and apply our current approach on an ArcticDEM strip dated 24 June 2018. Data are shown in Supplementary Figs. 6 and 7. Our method represents an advance on these previous approaches as it provides a direct measure of crevasse depth rather than simply area. While this also means the workflows are not quantiatively comparable (see ‘Limitations’ section), overall there is good qualitative agreement between the methods. Individual crevasses are identifiable between the three datasets. In comparison to the Sentinel-2 approach, our method is sensitive to smaller crevasses, as well as less likely to misclassify the edges of snow/ice boundaries. These advantages are balanced by the much higher temporal resolution of the Sentinel-2 stack, which can detect sub-seasonal changes^28^. Using the UAV data as ground validation, we assess the limit of crevasse width detectable by our method to be approximately 10 m. This matches the previous assessment made using a more rudimentary ArcticDEM segmentation approach^3^.

#### Limitations

The limitations of our dataset are derived from the resolution and optical source data of the raw ArcticDEM strips.

First, the 2 m resolution of the source strips places a fundamental lower bound on the minimum identifiable crevasse diameter. In practice, comparison with UAV data has shown that a realistic minimum diameter observable with these methods is ~10 m (see section ‘Uncertainty and method intercomparison’). Although this limits applications for smaller inland crevasses, it is more than sufficient for observation of changes at crevasse fields in fast-flowing (>100 m yr^−1^) regions, where the crevasse width averages ~60 m (see section ‘Determining the optimal crevasse length scale’).

Second, the reported crevasse depth values produced by our method are commonly in the range of 10–100 m deep. This does not represent full crevasse depth, as even crevasses with surface expressions of only tens of centimetres have been shown reach depths of hundreds of metres^65^. However, larger crevasses of the type observed in this study (tens of metres in width) have been observed to be consistently infilled with debris in high-resolution UAV-derived datasets^66^, limiting the observed depth in optically derived DEMs. As such, we refer to the volumetric measurements in this study as the ‘exposed crevasse air volume’, acknowledging that full-depth measurements are not possible. Full crevasse depths have been extrapolated from simpler 2D profiles in the past^21^, suggesting that a similar method to extrapolate 3D datasets may be possible in the future.

Third, the optical nature of the source data meant that we cannot extract snow-filled crevasses that may be possible to detect using other methods, such as synthetic aperture radar (SAR) or ground penetrating radar (GPR)^67^. However, the large diameters of crevasses detected here are highly unlikely to fill with snow: in analysis of Sentinel-2 optical imagery with a similar effective resolution for crevasse detection, crevasse density was not observed to change over a seasonal cycle or in an indicative elevation-dependent way that suggested snowfill^28^. The month filtering, ablation zone masking and median mosaicking we performed during the mosaicking process mean we consider it very unlikely that snowfill can explain any of the large-scale multitemporal change we observe in our study. Any small-scale variation should be adequately captured in our uncertainty assessment, alongside other minor sources of measurement variance (for example, satellite geometry).

Fourth, by selecting a relatively shallow BTH threshold of 1 m, we implicitly included features that are not true crevasses (for example, shallow ditches and river gulleys). We chose to do this as we are interested in volumetric change rather than area change, and these shallow features do not represent substantial contributions to aggregate volume measurements. Increasing the BTH threshold to a higher value introduces a much larger volume of false negatives instead of a small volume of false positives. Experimentation showed that increasing the threshold for crevasse identification may aesthetically improve the binary crevasse mask, but resulted in an increased variance in our volumetric uncertainty measurements as legitimate crevasses began to be inconsistently masked from DEM strips. As a result, we do not recommend our method for crevasse area segmentation tasks. Other methods have previously been proposed for this task using ArcticDEM^3^.

Finally, our analysis covers only the years 2016 and 2021, rather than a continuous dataset over the study period. Owing to limitations of data coverage in the ArcticDEM strip dataset, it is not possible to achieve satisfactory coverage of other years at a Greenland scale. We make the assumption that crevasses represent the ‘damped’ expression of multi-annual ice dynamics, and so assessing change between these years is valid as there is a negligible chance that changes we detect may be a result of capturing high interannual variability or measurement error. To show this, we extract 2016–2021 annual crevasse volume at six select Greenlandic outlets where data availability is sufficient: three where substantial acceleration occurs over the time period (Anorituup Kangerlua fjord, KIV Steenstrups and Kjer Glacier); and three where stable or decelerating trends are prevalent (Umiammakku Sermiat, SKSG and Rink Isbræ) (Supplementary Fig. 2). We overlay ice velocity from ITS_LIVE data and, for Anorituup Kangerlua fjord, also present individual mosaics for further reference (Supplementary Fig. 8). These data support our assumption that interannual variation is low and dynamic response occurs on timescales greater than one year (for example, KIV Steenstrups and Kjer Glacier both continue to increase in volume in 2021 despite peaking in velocity in 2020), and align with previous studies on this topic^44,68^. Additionally, the secular trends in crevasse volume are clearly associated with parallel increases and/or decreases in glacier velocity. This supports our inference that observed changes are attributable to real changes in crevasse volume rather than short-term variability or measurement error.

### Discharge

We compared crevasse change to discharge change as a proxy for the bulk dynamic change of ice sectors and basins. This assumes that the time-evolving discharge, ice velocity and the magnitude/extent of extensional stress are broadly correlated at a basin and sectoral scale. Furthermore, as discharge is a function of both ice velocity and outlet size, comparing bulk crevasse volume to bulk discharge implicitly controlled for available ice surface area, unlike direct measurements of ice flow velocity or strain rates.

Changes in dynamic forcing take time to propagate through to observed changes in crevasse fields, as crevasses are the cumulative product of opening and closing stresses integrated over time. Over the majority of the ice sheet, these strain rates are of the order of 0.01 per year or less^69^, hence changes in crevasse width cannot fluctuate more than a few per cent in a given year and changes will be dominated by multi-year trends in flow. This is evidenced by low interannual trends and long-term secular trends of the order of years observed in previous studies^28,30^. A period of five years was selected to be a reasonable estimate of crevasse response time in line with published estimates of crevasse life cycles in studies of valley glaciers^44,68^ and ensured discharge records do not overlap. As a result, we compared the average annual discharge for the preceding five years (2012–2016 for the 2016 crevasse dataset and 2017–2021 for the 2021 dataset).

We obtained 2012–2021 monthly ice discharge measurements from flux gate measurements at marine-terminating glaciers from two complementary datasets^39,40^ (hereafter the ‘King’ and ‘Mankoff’ datasets). Errors presented here were propagated from those reported in these source datasets. Each individual dataset covers specific outlet glaciers, and neither is comprehensive across all Greenland outlets. As the pre-defined drainage basins^50^ frequently contain multiple outlets, any individual drainage basin may be comprehensively covered by flux gates from either the King or Mankoff datasets, both or neither. As a result, we combined the datasets to cover as many discharge basins as possible. Of the 254 basins in the dataset, we assessed 192 as having discharge records in at least one dataset and, of these, 185 basins were usable. Of the 185 usable basins, 138 had outlets comprehensively covered by both King and Mankoff, so we took the average of the two datasets. A further 29 and 16 basins were comprehensively covered only by King or Mankoff, respectively. At two basins, unusually, the two datasets covered mutually exclusive outlets within the basin, and we used the sum of the two datasets to represent full basin discharge.

## Online content

Any methods, additional references, Nature Portfolio reporting summaries, source data, extended data, supplementary information, acknowledgements, peer review information; details of author contributions and competing interests; and statements of data and code availability are available at 10.1038/s41561-024-01636-6.

## Supplementary information

Supplementary Information Supplementary Figs. 1–8 and Supplementary Tables 2 and 3.

Supplementary Table 1 Sectoral and total crevasse volumes. Data for 2016, 2021 and the change between 2016 and 2021.

## Data Availability

Source data necessary to reproduce this study and the figures within (Greenland-wide crevasse volume rasters, and basin-scale aggregations of crevasse volume and discharge) are available via Figshare at 10.6084/m9.figshare.27902268 (ref. ^70^). ArcticDEM 2 m strips are available at 10.7910/DVN/OHHUKH. The EIGEN-6C4 model is available as part of the BedMachine v4 at 10.5067/VLJ5YXKCNGXO. The Greenland Ice Mapping Project ice and ocean classification mask is available at 10.5067/B8X58MQBFUPA. Raw Mankoff discharge data are available at 10.22008/promice/data/ice_discharge.

## Code availability

The full workflow to download and extract crevasses from ArcticDEM and REMA imagery is publicly available as a Python package at https://github.com/trchudley/crevdem and via Zenodo at 10.5281/zenodo.14199587 (ref. ^71^).
