# Supplementary material for: Increased crevassing across accelerating Greenland Ice Sheet margins
Source: Nat Geosci. 2025 Feb 3;18(2):148–53. doi: 10.1038/s41561-024-01636-6 (PMC11810776; doi:10.1038/s41561-024-01636-6)
Supplement: Supplementary file 1 — Supplementary Figs. 1–8 and Supplementary Tables 2 and 3. [file 41561_2024_1636_MOESM1_ESM.pdf]

nature geoscience

Supplementary information https://doi.org/10.1038/s41561-024-01636-6

Increased crevassing across accelerating Greenland Ice Sheet margins

In the format provided by the
authors and unedited

Table of Contents

- Supplementary Figures 1 – 8
- Supplementary Tables 2 – 3

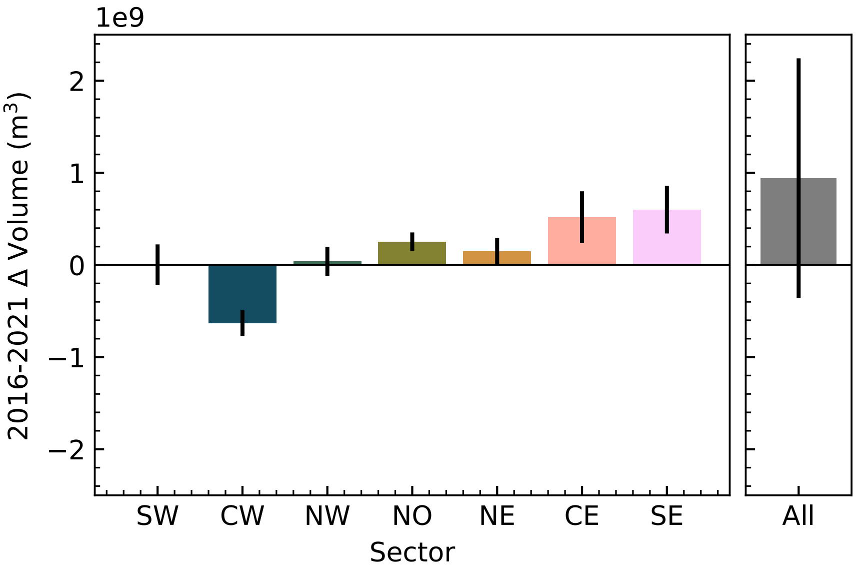

Supplementary Fig. 1: Crevasse volume change at an ice-sheet-wide scale. Presented as for Figure 2d, but with the ice-sheet-wide value for scale. Error bars represent 2σ uncertainties (see Methods).

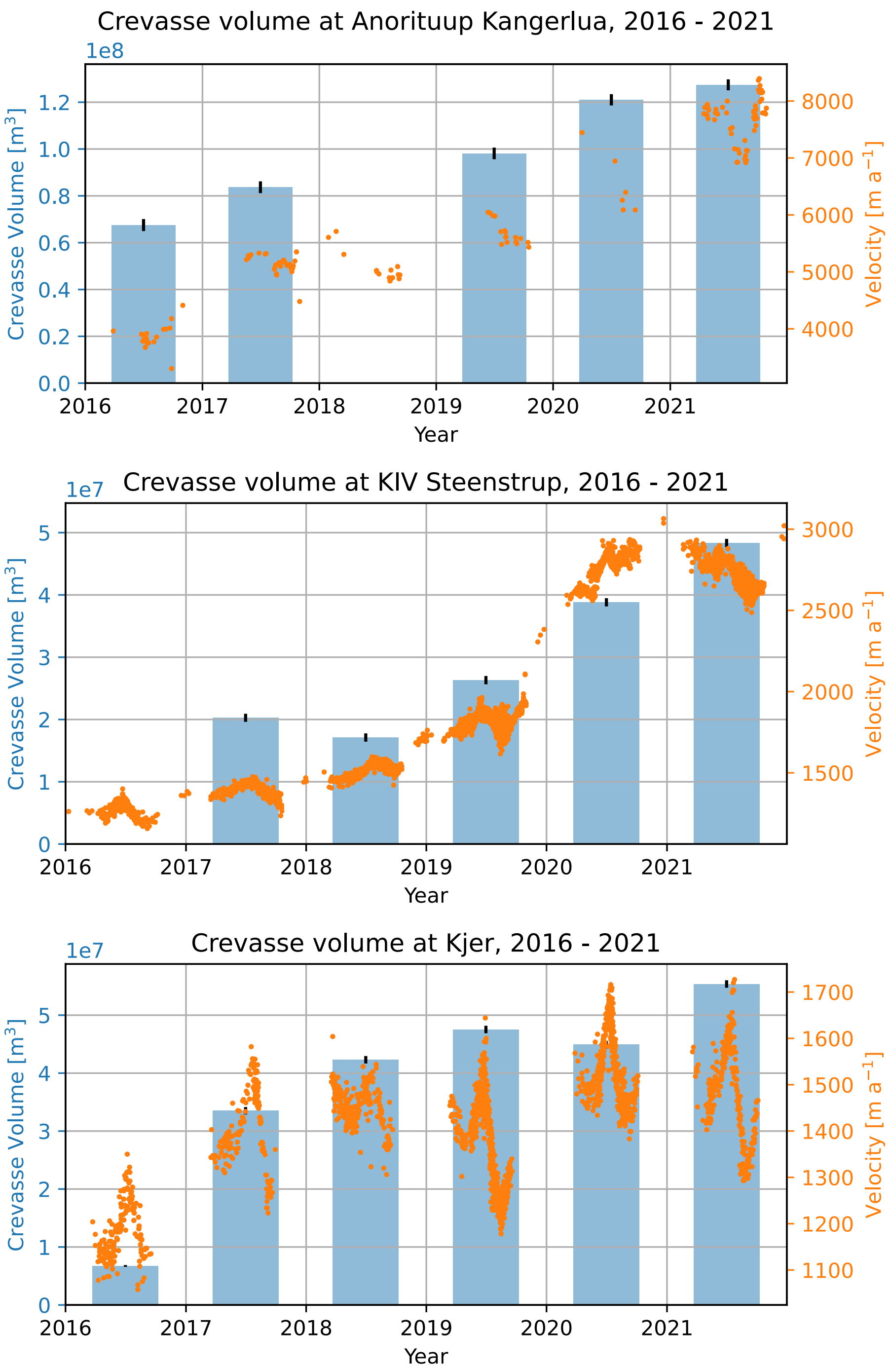

Supplementary Fig. 2: Annual crevasse volume and ITS-LIVE velocities at selected glaciers. Presented for (top) Anorituup Kangerlua ; (middle) KIV Steensups Nordre Bræ; and (bottom) Kjer Glacier. Blue bars indicate the annual average crevasse volume for 2016 through to 2021. Error bars represent 2σ uncertainties (see Methods). Orange dots indicate optically-derived ITS-LIVE data (filtered to time steps of ≥16 days) at the trunks of the glaciers between 2016 and 2021. Location bounds and data are provided in supplementary data.

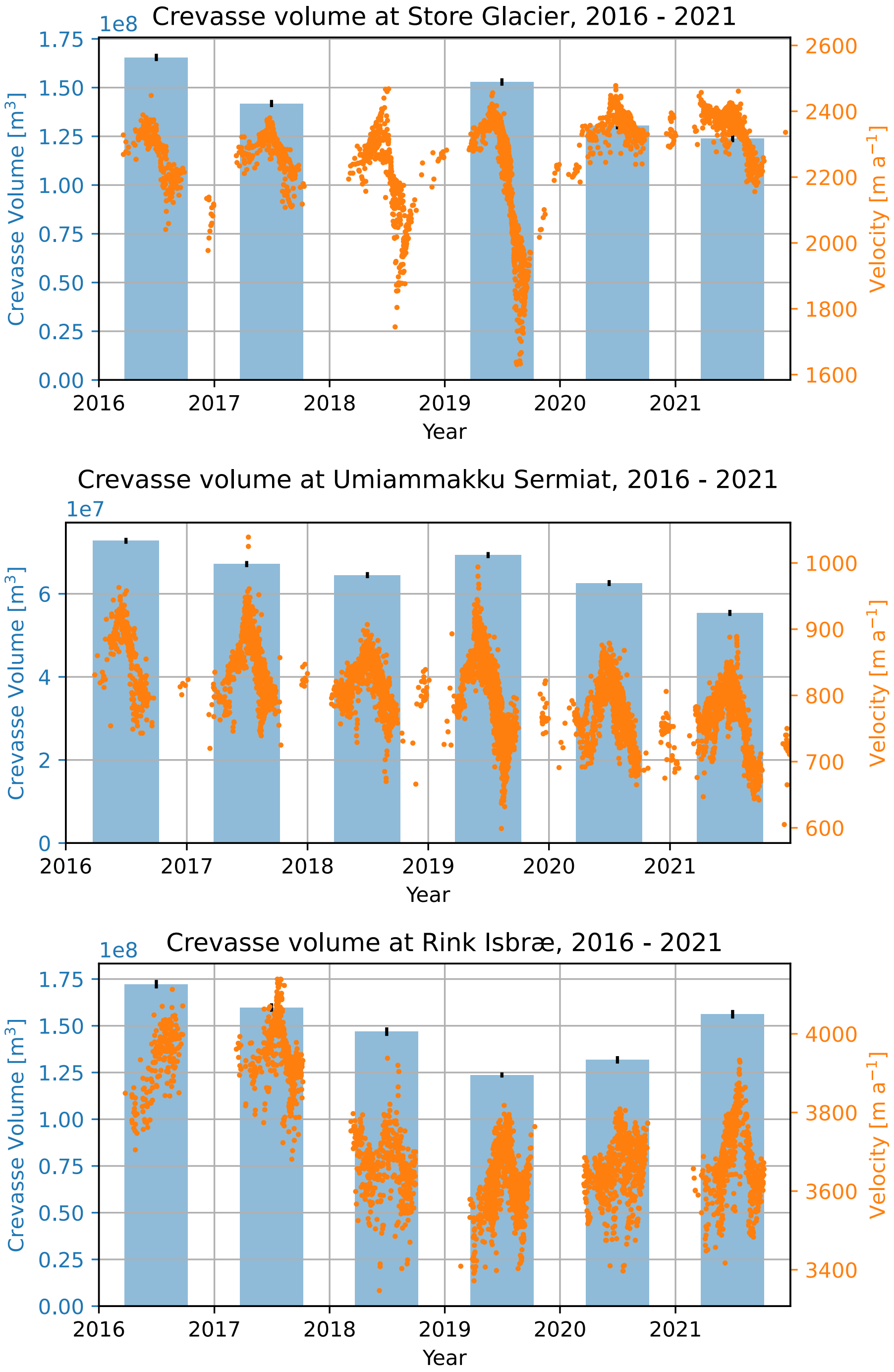

Supplementary Fig. 2 (continued): Annual crevasse volume and ITS-LIVE velocities at selected glaciers. Presented for (top) Store Glacier; (middle) Umiammakku Sermiat; and (bottom) Rink Isbræ.

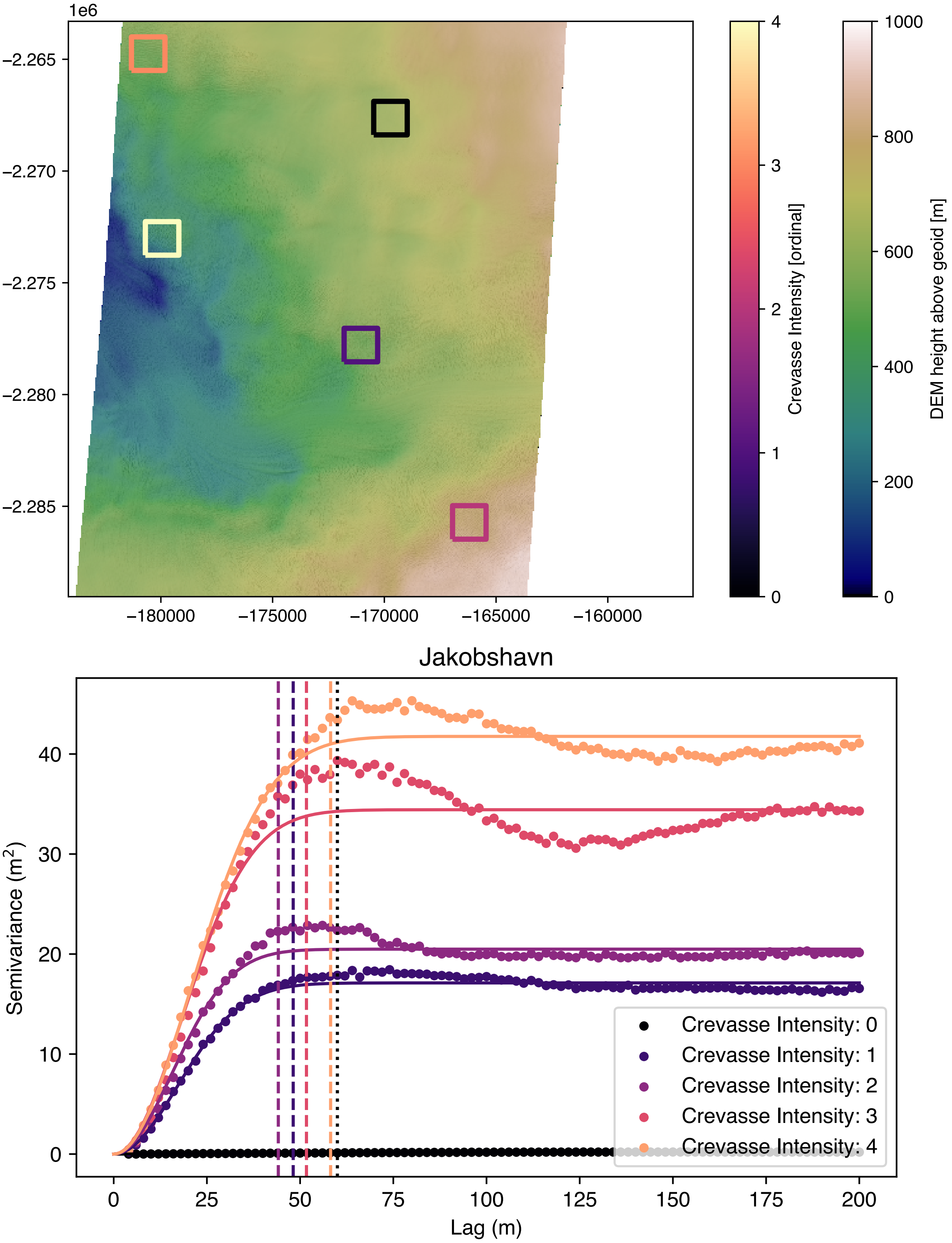

Supplementary Fig. 3a: Variogram analysis at Sermeq Kujalleq (Jakobshavn Isbræ). Top: Map of sample ArcticDEM strip at Sermeq Kujalleq (Jakobshavn Isbræ), with identified sample zones outlined in red and coloured according to qualitative ordinal crevasse intensity. Coordinates in NSIDC Polar Stereographic North. Bottom: Semivariograms from sample zones, with modelled range as vertical dashed lines and final length scale (60 m) as vertical black dotted line.

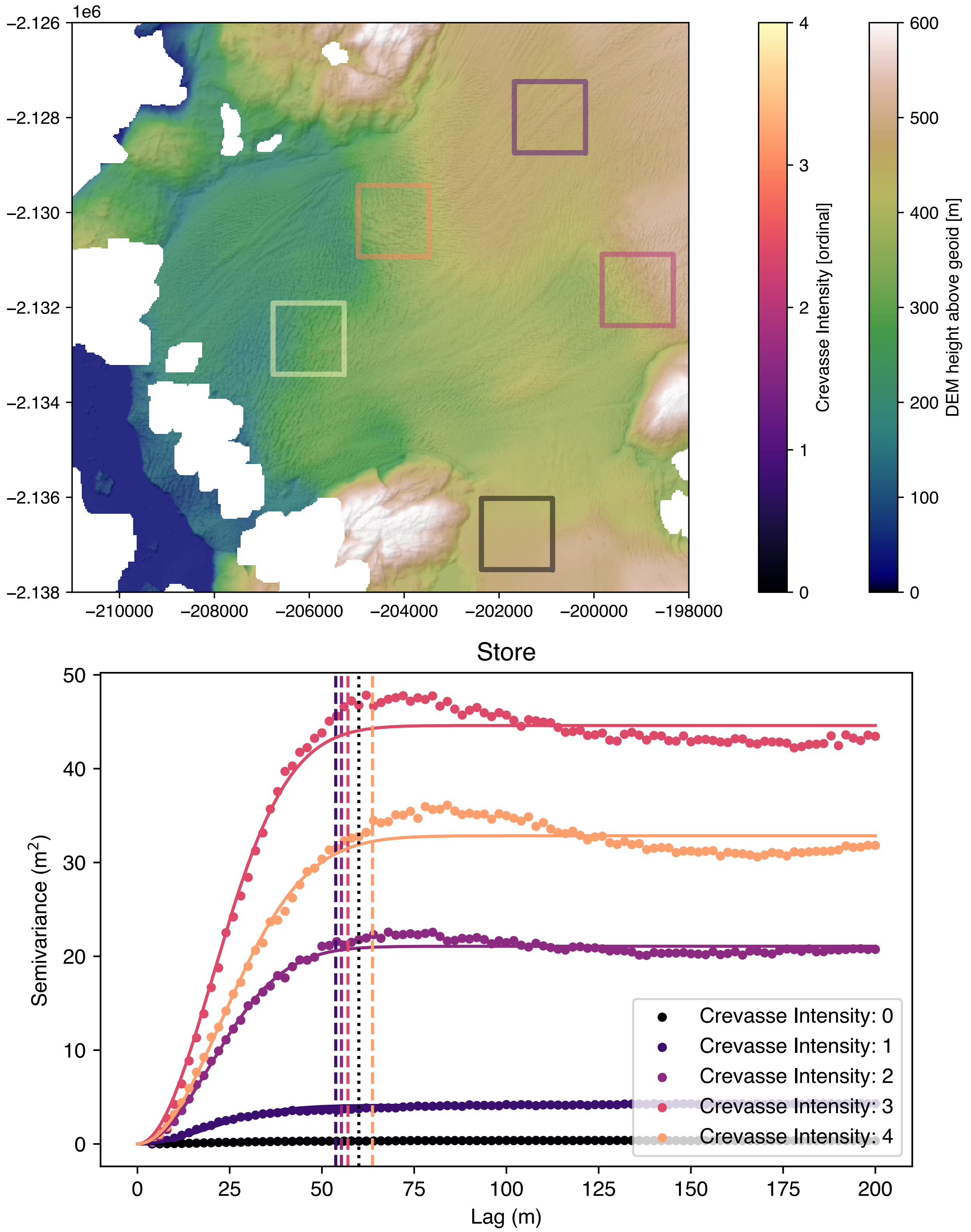

Supplementary Fig. 3b: Variogram analysis at Sermeq Kujalleq (Store Glacier). Top: Map of sample ArcticDEM strip at Sermeq Kujalleq (Store Glacier), with identified sample zones outlined in red and coloured according to qualitative ordinal crevasse intensity. Coordinates in NSIDC Polar Stereographic North. Bottom: Semivariograms from sample zones, with modelled range as vertical dashed lines and final length scale (60 m) as vertical black dotted line.

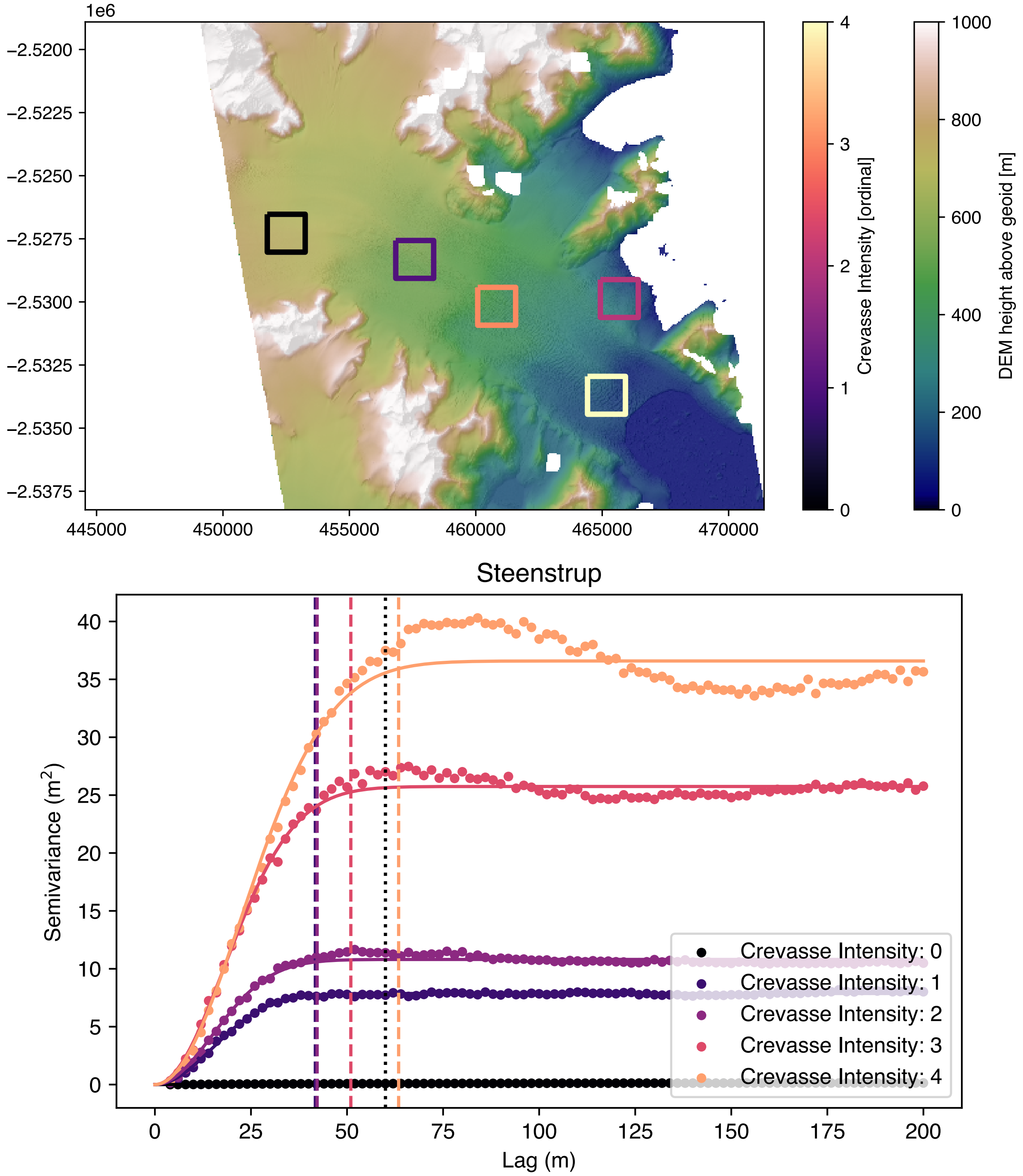

Supplementary Fig. 3c: Variogram analysis at KJV Steenstrups Nordre Bræ. Top: Map of sample ArcticDEM strip at KJV Steenstrups Nordre Bræ, with identified sample zones outlined in red and coloured according to qualitative ordinal crevasse intensity. Coordinates in NSIDC Polar Stereographic North. Bottom: Semivariograms from sample zones, with modelled range as vertical dashed lines and final length scale (60 m) as vertical black dotted line.

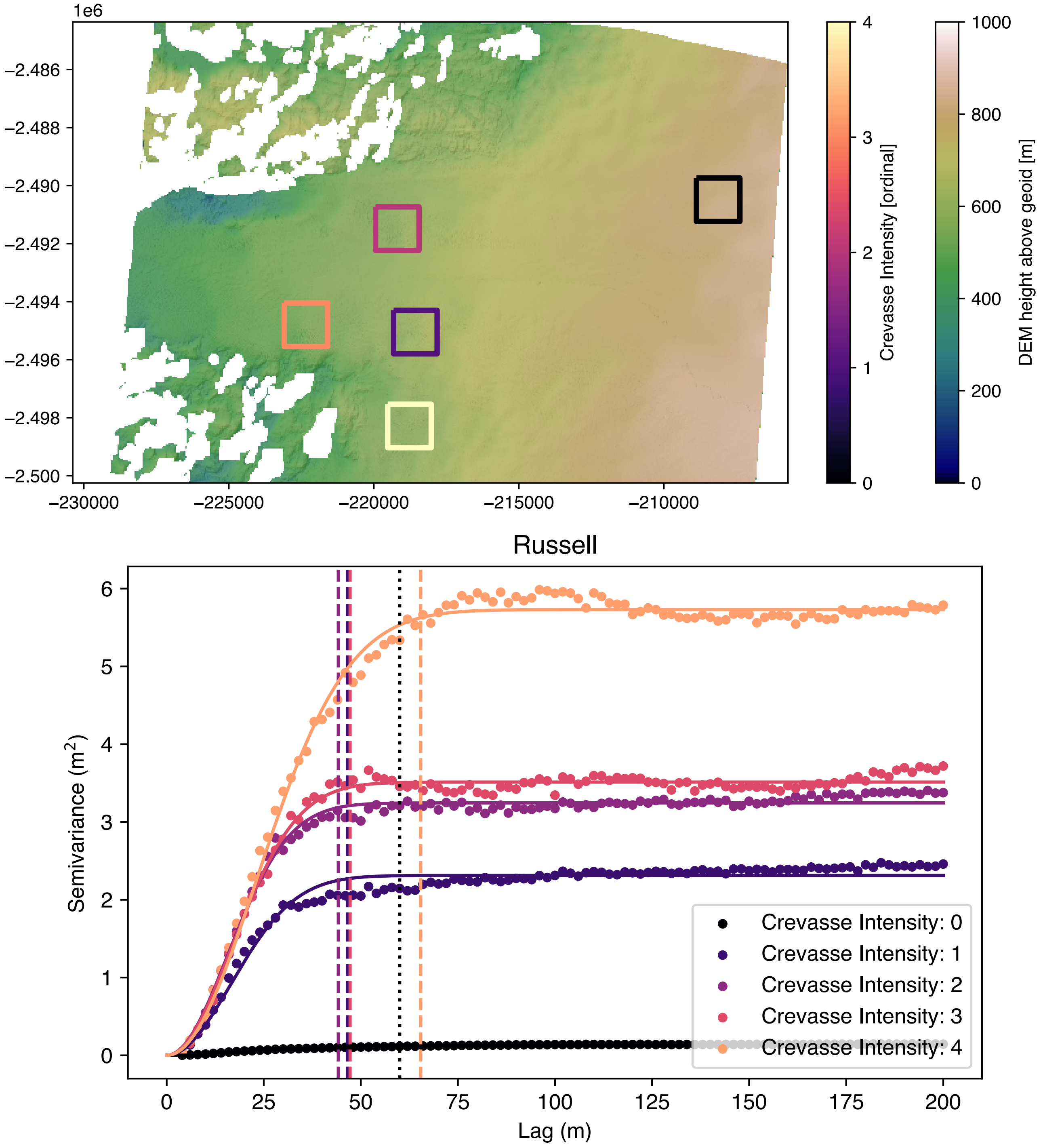

Supplementary Fig. 3d: Variogram analysis at Isunnguata Sermia. Top: Map of sample ArcticDEM strip at Isunnguata Sermia, with identified sample zones outlined in red and coloured according to qualitative ordinal crevasse intensity. Coordinates in NSIDC Polar Stereographic North. Bottom: Semivariograms from sample zones, with modelled range as vertical dashed lines and final length scale (60 m) as vertical black dotted line.

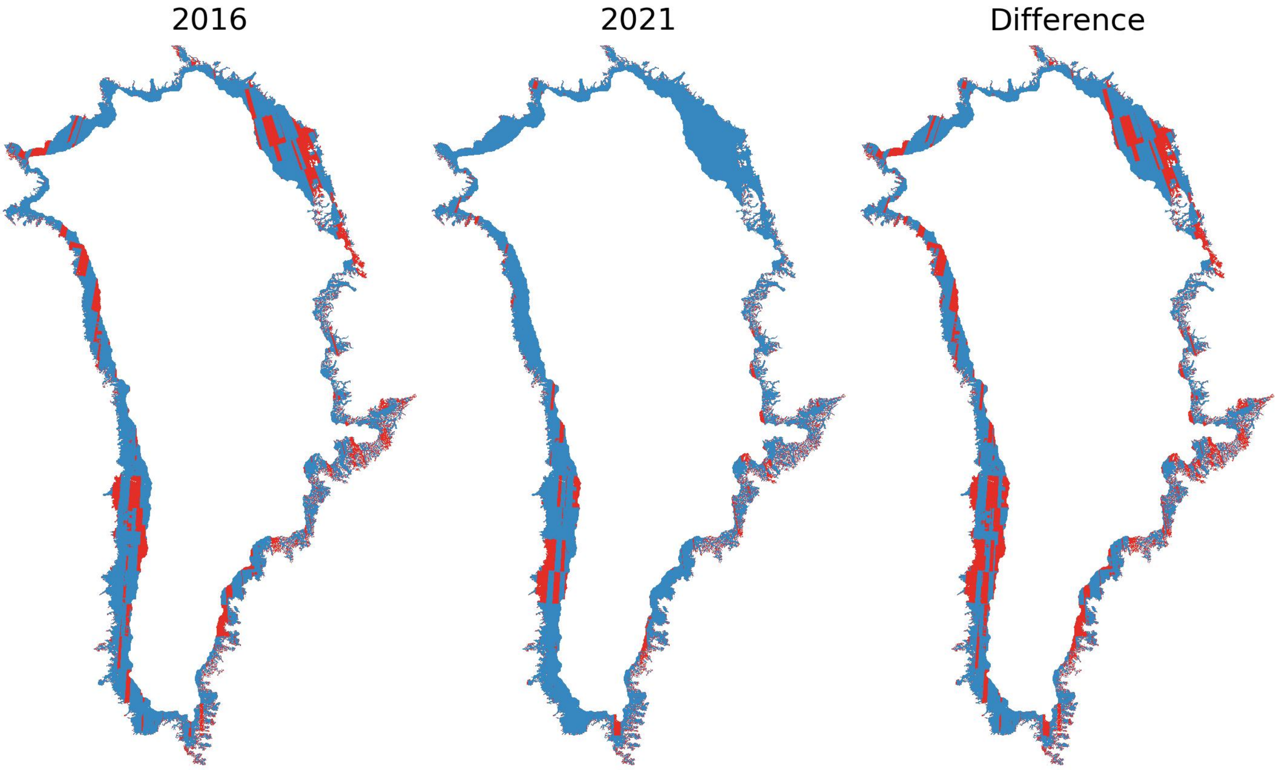

Supplementary Fig. 4: Maps of crevasse data coverage. Regions of coverage are in blue and absence is in red. (a) 2016; (b) 2021; (c) 2016-2021.

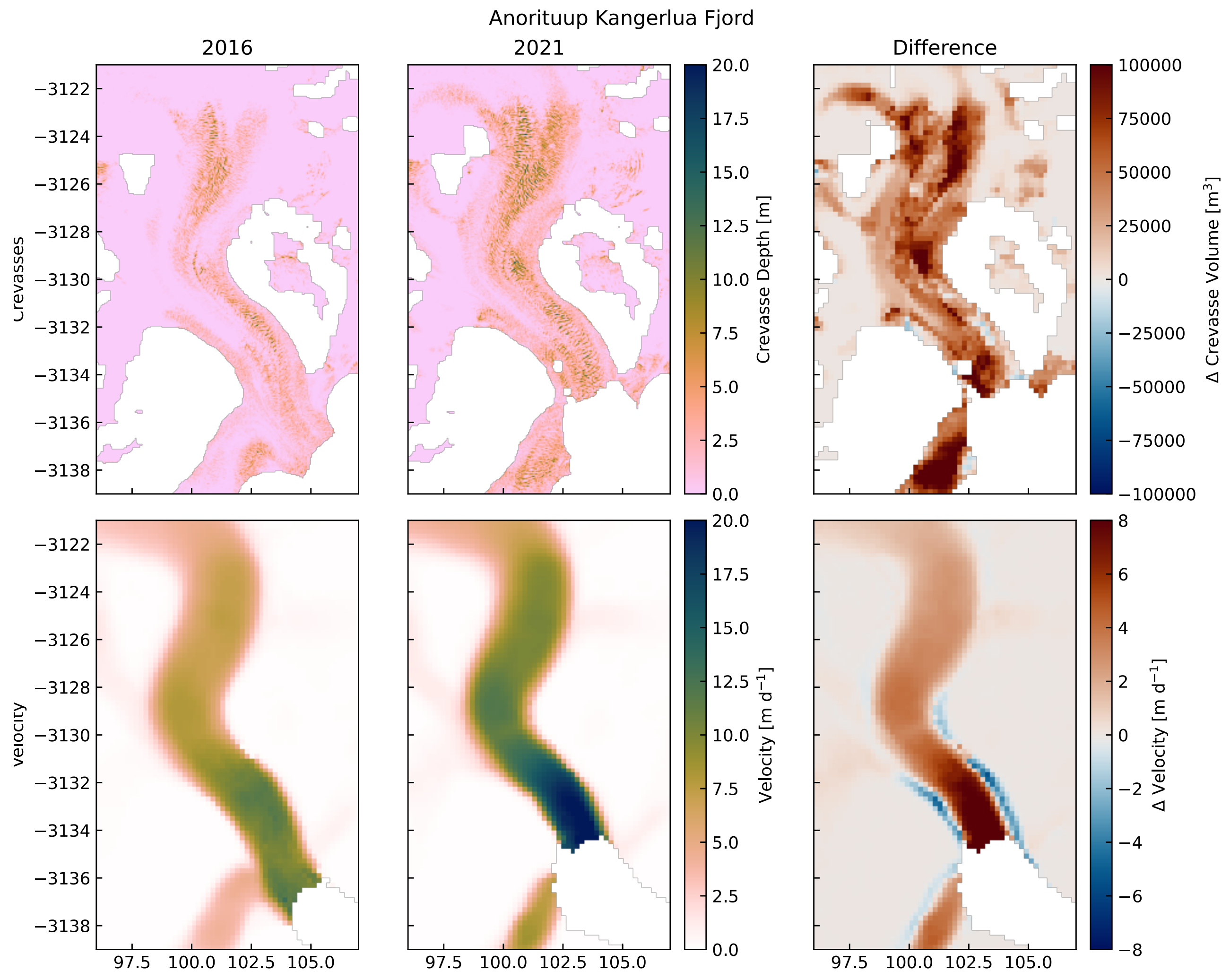

Supplementary Fig. 5a: Example of crevasse and velocity changes at selected glaciers. Glacier change shown at Anorituup Kangerlua Fjord. Top row, left to right: (left) 2 m mosaic of crevasse distribution in 2016. (middle) 2 m mosaic of crevasse distribution in 2021. (right) Change in crevasse volume, at 200 m resolution, between 2016 and 2021. Bottom row, left to right: (left) Annual velocity mosaic for 2016. (middle) Annual velocity mosaic for 2021. (right) Change in annual velocity between 2016 and 2021. Velocity mosaics are MEaSUREs Greenland annual ice sheet velocity mosaics [1, 2].

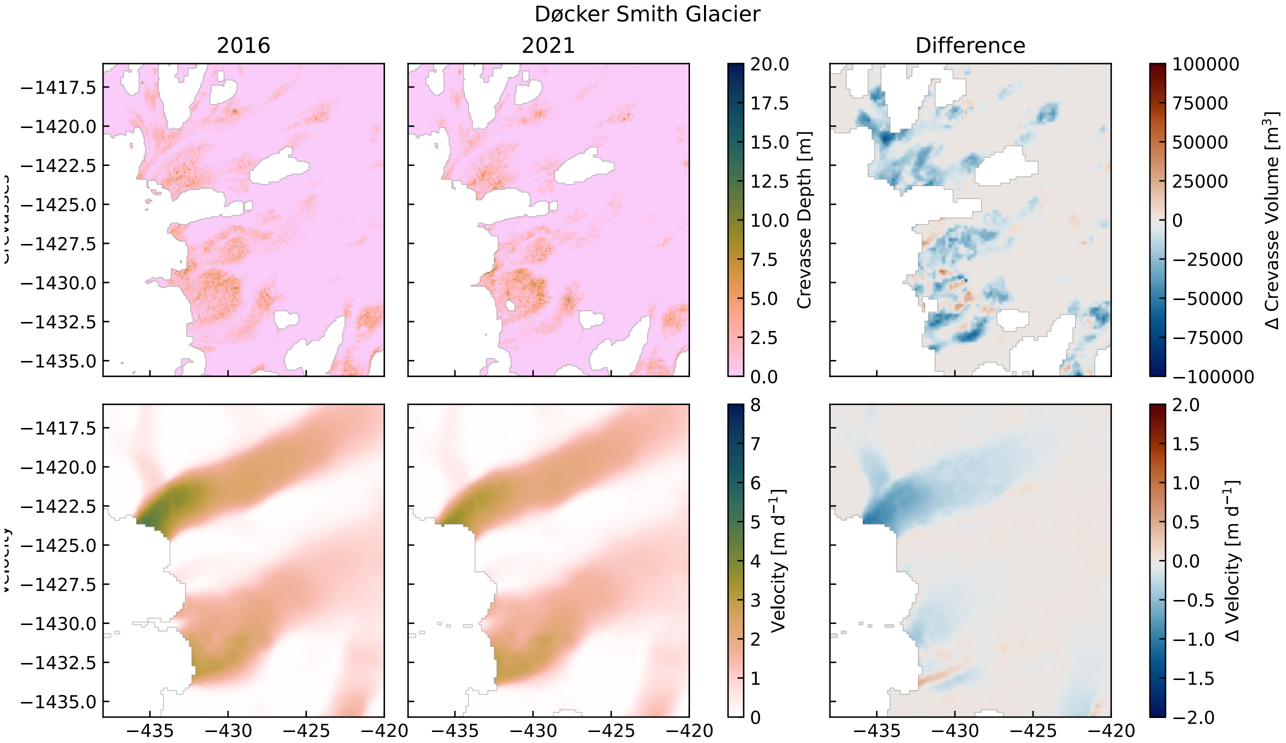

Supplementary Fig. 5b: Example of crevasse and velocity changes at selected glaciers. Glacier change shown at Døcker Smith Glacier. Panels align with those described in the caption of Figure 5a.

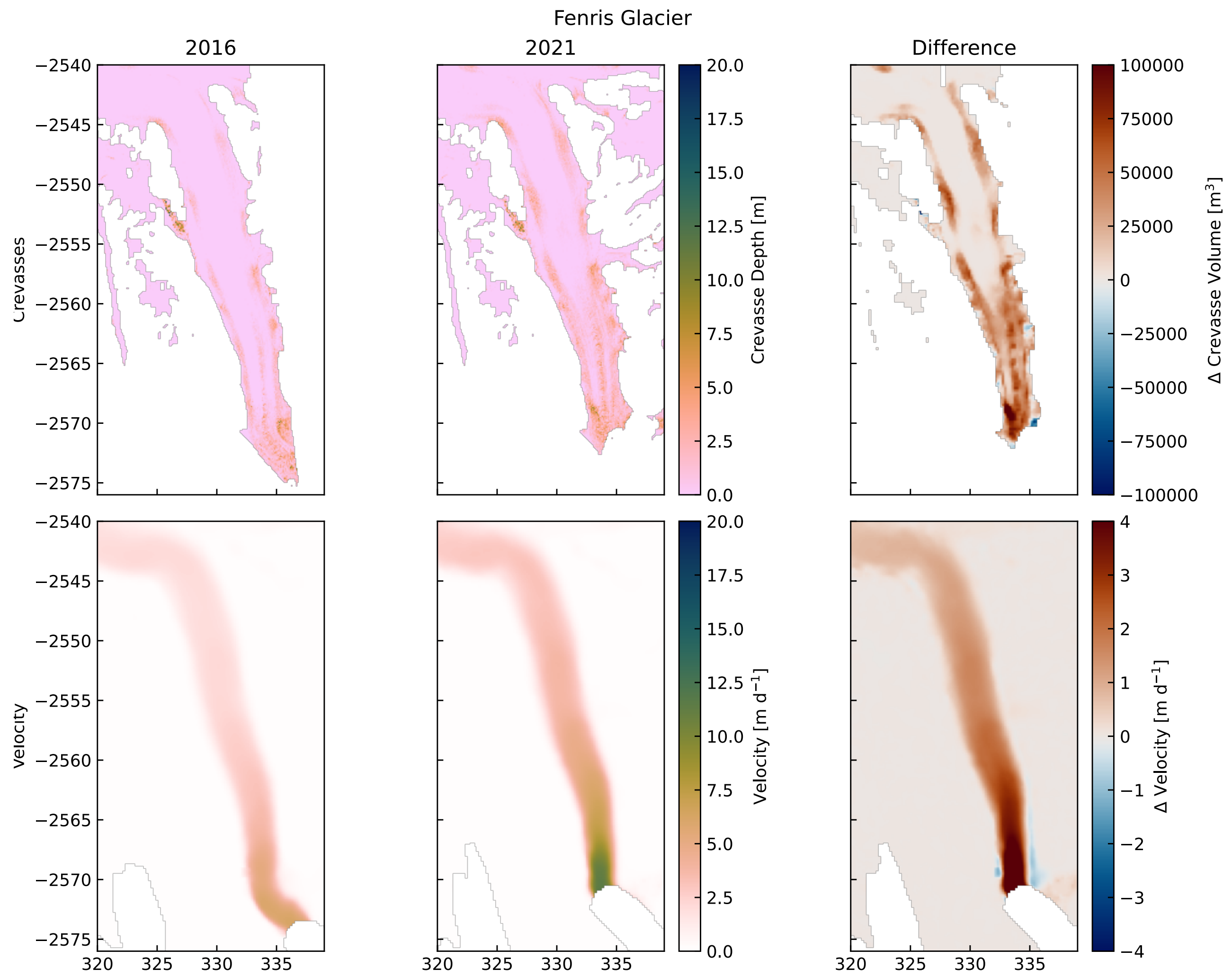

Supplementary Fig. 5c: Example of crevasse and velocity changes at selected glaciers. Glacier change shown at Fenris Glacier. Panels align with those described in the caption of Figure 5a.

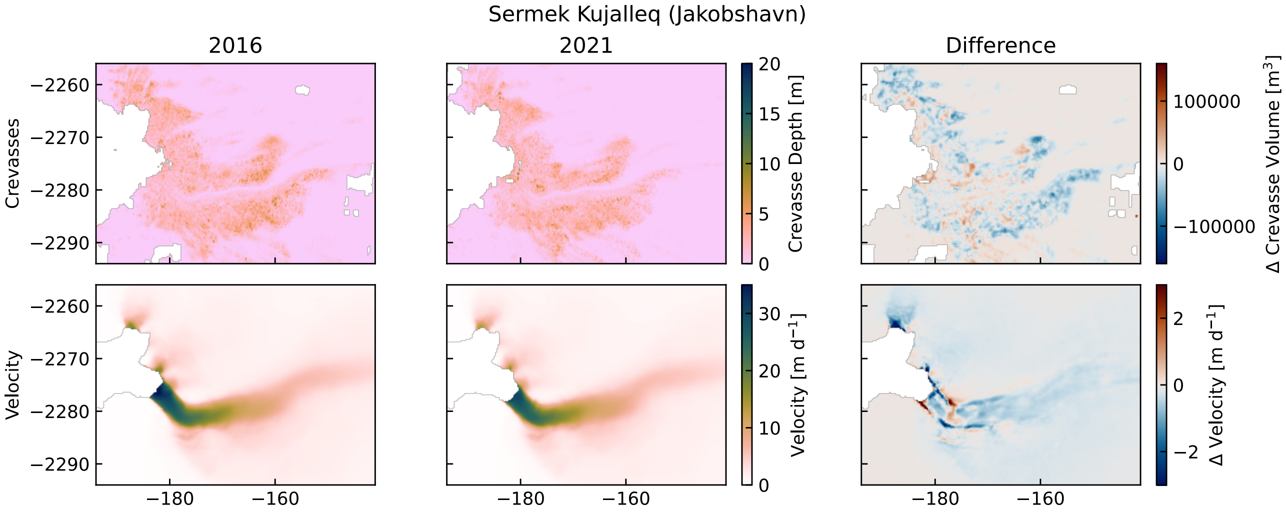

Supplementary Fig. 5d: Example of crevasse and velocity changes at selected glaciers. Glacier change shown at Sermeq Kujalleq (Jakobshavn Isbræ). Panels align with those described in the caption of Figure 5a.

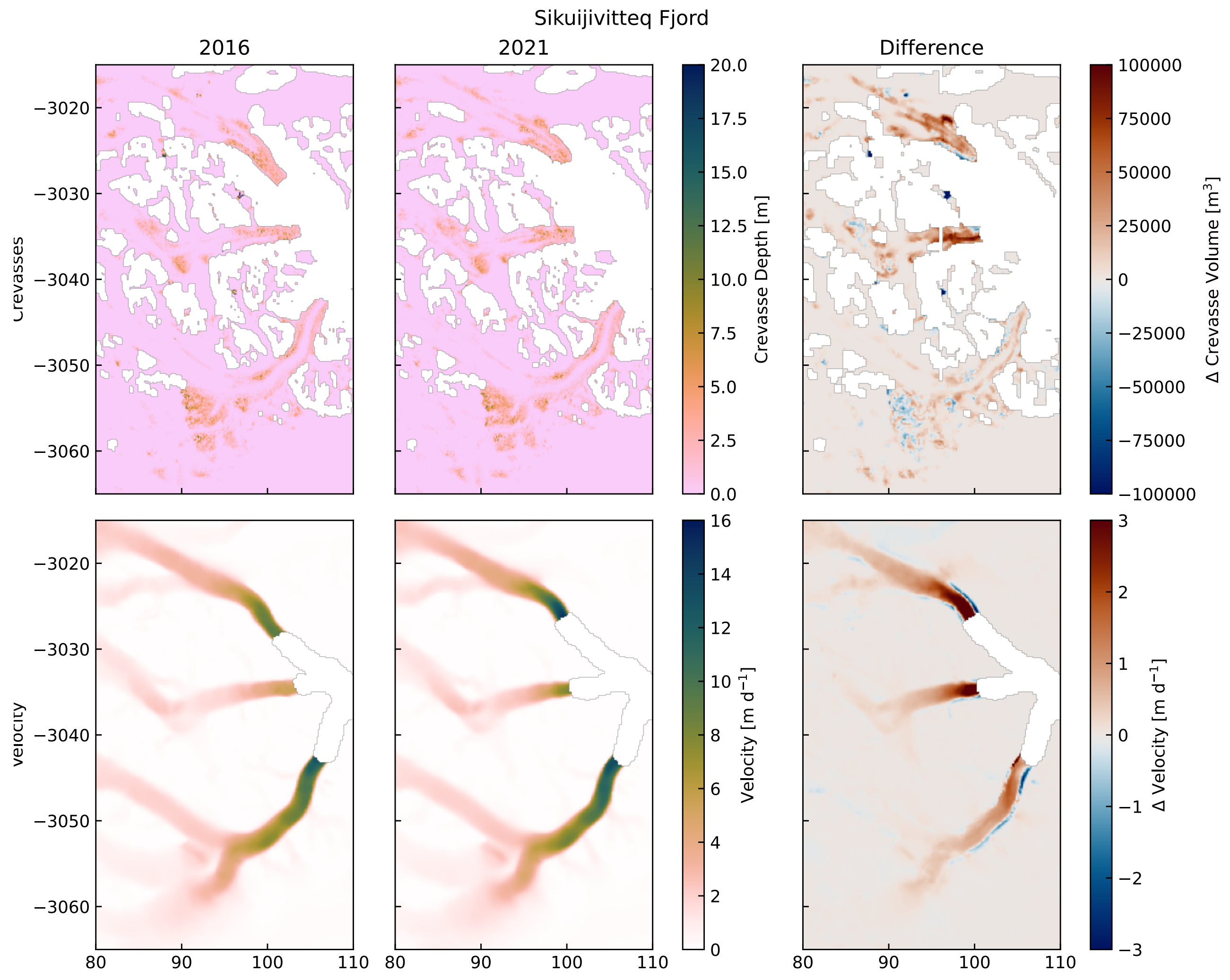

95 **Supplementary Fig. 5e: Example of crevasse and velocity changes at selected glaciers.** Glacier change shown at Sikuijivitteq Fjord. Panels align with those described in the caption of Figure 5a.

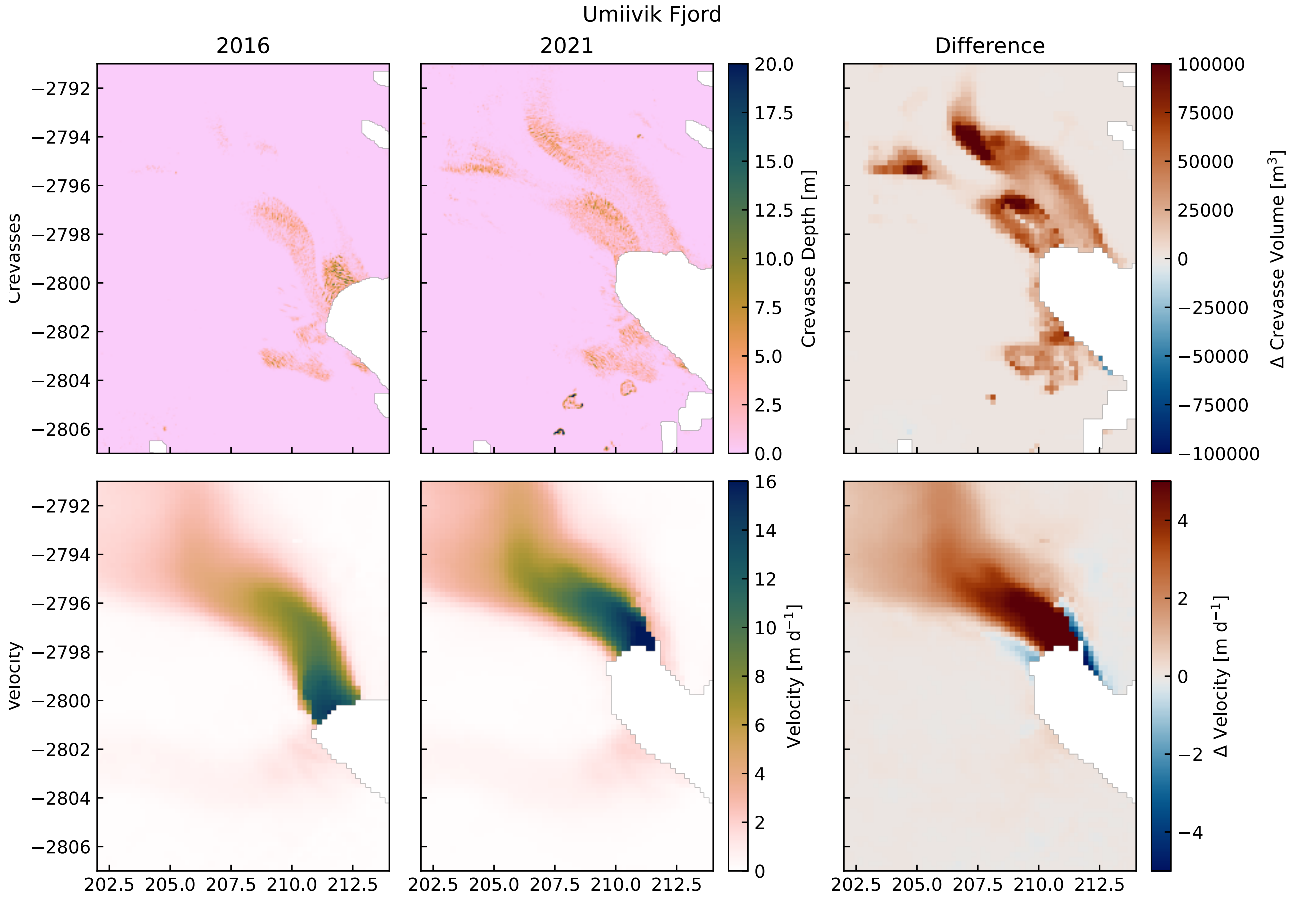

100 **Supplementary Fig. 5f: Example of crevasse and velocity changes at selected glaciers.** Glacier change shown at Umiivik Fjord. Panels align with those described in the caption of Figure 5a.

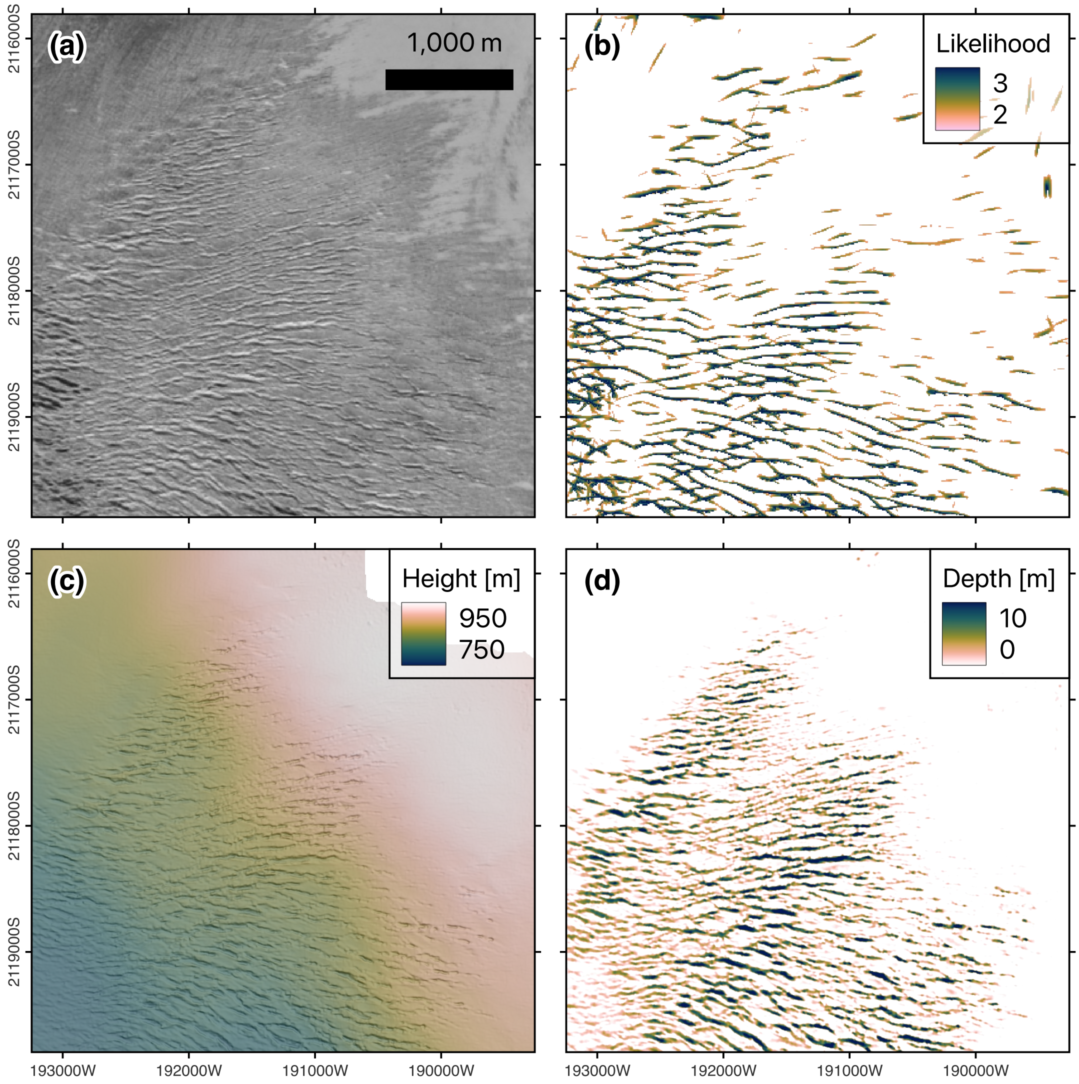

Supplementary Fig. 6: Comparison between crevasse detection methods at Store Glacier crevasse field. (a) Band 2 Sentinel-2 image of Store Glacier crevasse field, 2018-07-02. (b) Crevasse detection from Sentinel-2 data following optical extraction method [3]. (c) ArcticDEM strip of Store Glacier crevasse field, 2018-06-24. (d) Crevasse detection from ArcticDEM strip following the method described in this study. Axis labels refer to coordiantes in EPSG:3413.

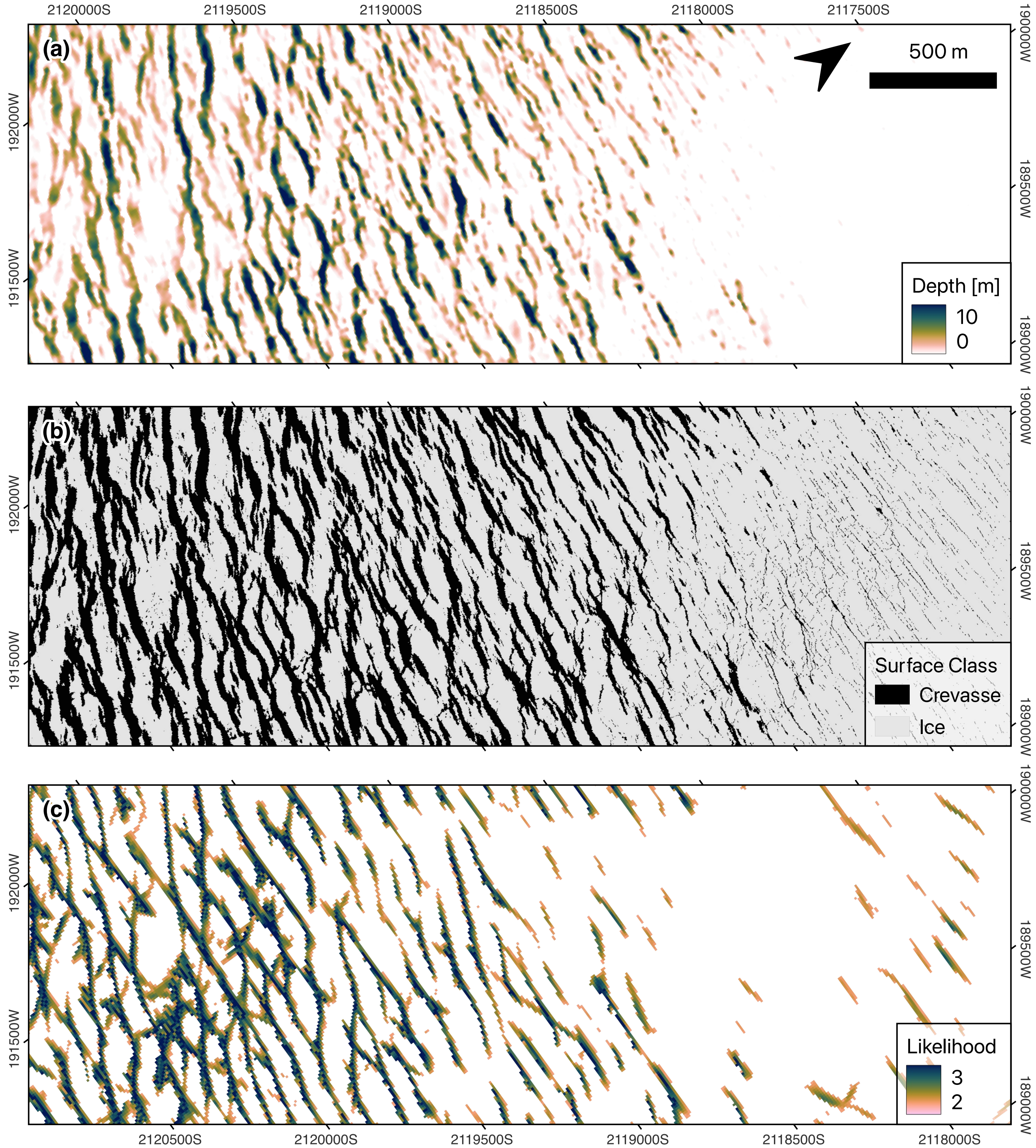

Supplementary Fig. 7: Comparison between crevasse detection methods at Store Glacier crevasse field. (a) Crevasse detection from ArcticDEM strip dated 2018-06-24, following the method described in this study. (b) Crevasse detection from UAV-derived data at 15 cm resolution following object-based machine learning techniques [4]. (c) Crevasse detection from Sentinel-2 data dated 2018-07-02 following optical extraction method [3]. Axis labels refer to coordinates in EPSG:3413.

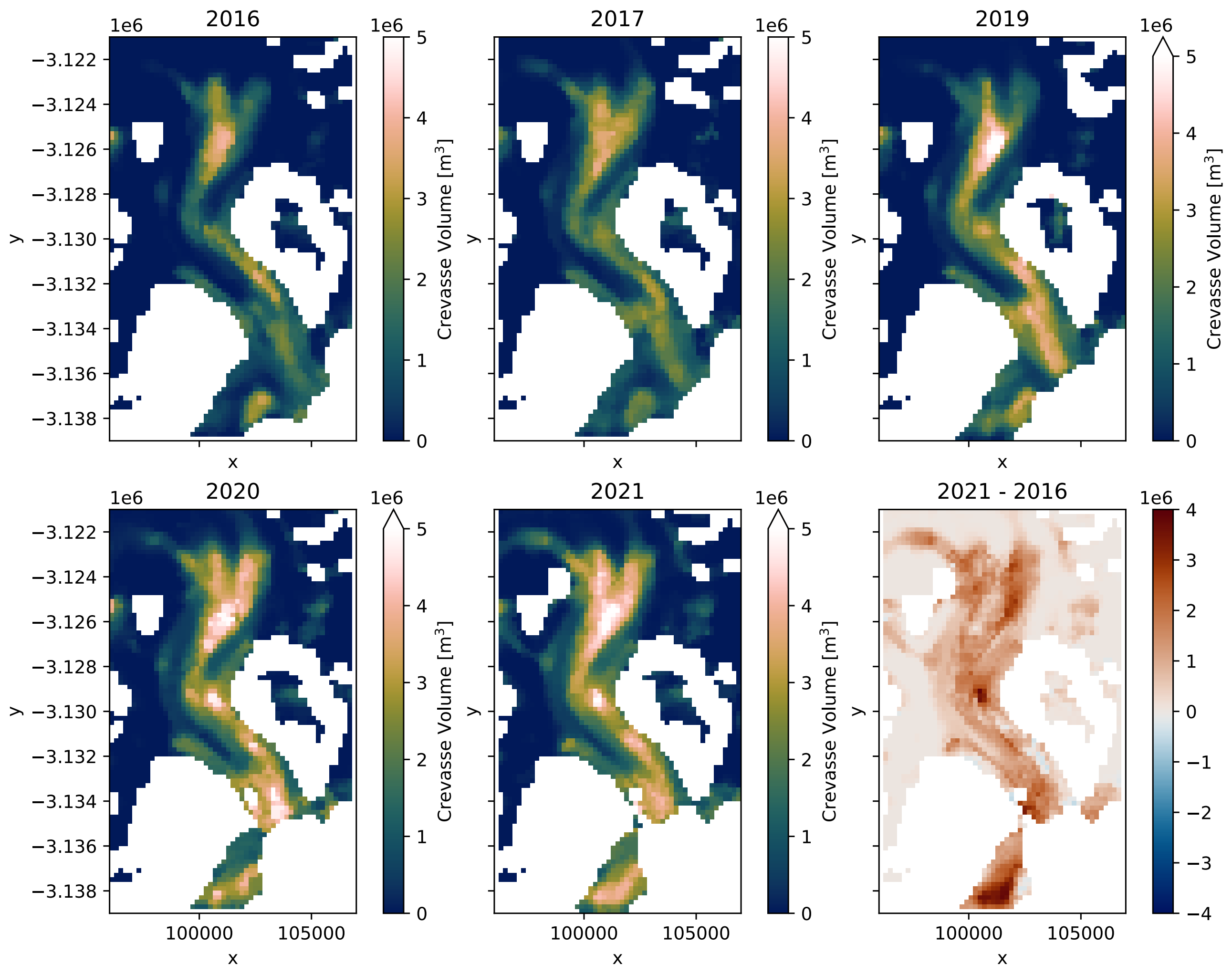

Supplementary Fig. 8: Evolution of crevasse fields at glacier at head of Anorituup Kangerlua Fjord. Crevasse volume, at 200 m resolution, in (top left) 2016; (top middle) 2017; (top right) 2019; (bottom left) 2020; and (bottom middle) 2021. (Bottom right) Change in crevasse volume, at 200 m resolution, between 2016 and 2021. Axis labels refer to coordinates in EPSG:3413.

Supplementary Table 2: Full results of regression analyses at sector- and basin-scale, including with and without outlier basins identified in the main text.

Scale	Correlation	Including outlier basins? (SKJI, SKSG, HMB)	p	R^2	RMSE (m^3)
**Sector**	Discharge : Crevasse volume	True	0.04	0.60	9.89E+08
		False	0.04	0.60	9.76E+08
	Δ Discharge : Δ Crevasse volume	True	0.00	0.97	5.33E+07
		False	0.01	0.78	9.83E+07
**Basin**	Discharge : Crevasse volume	True	0.00	0.64	9.66E+07
		False	0.00	0.50	9.68E+07
	+ve Δ Discharge : Δ Crevasse volume	True	0.00	0.20	3.38E+07
		False	0.00	0.29	2.62E+07
	-ve Δ Discharge : Δ Crevasse volume	True	0.46	0.01	1.47E+07
		False	0.65	0.00	1.44E+07

Supplementary Table 3: Number of ArcticDEM strips processed for each sector.

**Region**	**2016**	**2021**
**Southeast**	1361	1253
**Southwest**	1331	966
**Northeast**	496	1075
**Northwest**	1588	913
**Total**	**4776**	**4207**

References

1. Joughin, I. MEaSUREs Greenland Ice Velocity Annual Mosaics from SAR and Landsat, Version 5. NASA National Snow and Ice Data Center DAAC https://doi.org/10.5067/USBL3Z8KF9C3 (2023).
2. Joughin, I., Smith, B. E., Howat, I. M., Scambos, T. & Moon, T. Greenland flow variability from ice-sheet-wide velocity mapping. *Journal of Glaciology* **56**, 415–430 (2010).
3. Van Wyk de Vries, M., Lea, J. M. & Ashmore, D. W. Crevasse density, orientation and temporal variability at Narsap Sermia, Greenland. *Journal of Glaciology* 1–13 (2023) doi:10.1017/jog.2023.3.
4. Chudley, T. R. *et al.* Controls on Water Storage and Drainage in Crevasses on the Greenland Ice Sheet. *Journal of Geophysical Research: Earth Surface* **126**, e2021JF006287 (2021).
